# A computer-controlled data acquisition and analysis system for use in the characterization of plasma atomic emission sources

**DOI:** 10.1155/S1463924691000251

**Published:** 1991

**Authors:** James P. Shields, Edward H. Piepmeier

**Affiliations:** 1 Department of Chemistry Oregon State University Corvallis Oregon 97331 USA; 2 Hewlett-Packard Company Ink-Jet Components Division 1020 NE Circle Blvd Corvallis Oregon 97330 USA

## Abstract

The characterization of new spectroscopic plasma sources for use in atomic emission spectroscopy often presents the need for information about the spatial emission characteristics of the source. In addition, information about the variation of the emission over time is often beneficial. It is also useful to be able to graphically see, in real-time, the effects of varying a particular experimental parameter. This paper describes a flexible data acquisition system in which an echelle spectrometer is interfaced to an IBM-PC compatible microcomputer. The FORTH-based ASYST software, which proves real-time display of acquired data, allows any point in the emission source to be imaged on the entrance slit of the spectrometer by using the cursor keys of the PC and a computer-controlled mirror. The different acquisition modes of the system are illustrated with examples of two-dimensional spatial profiles of the plasma and monitoring of plasma stability over time. In addition, an illustration of the optimization of the echelle's aperture plate and photomultiplier tube positions in the focal plane is presented.


Journal of Automatic Chemistry, Vol. 13, No. 4 (July–August 1991), pp. 129–137

### A computer-controlled data acquisition and analysis system for use in the characterization of plasma atomic emission sources

**James P. Shields* and Edward H. Piepmeier†**
*Department of Chemistry, Oregon State University, Corvallis, Oregon 97331, USA.*

*The characterization of new spectroscopic plasma sources for use in atomic emission spectroscopy often presents the need for information about the spatial emission characteristics of the source. In addition, information about the variation of the emission over time is often beneficial. It is also useful to be able to graphically see, in real-time, the effects of varying a particular experimental parameter. This paper describes a flexible data acquisition system in which an echelle spectrometer is interfaced to an IBM-PC compatible microcomputer. The FORTH-based ASYST software, which proves real-time display of acquired data, allows any point in the emission source to be imaged on the entrance slit of the spectrometer by using the cursor keys of the PC and a computer-controlled mirror. The different acquisition modes of the system are illustrated with examples of two-dimensional spatial profiles of the plasma and monitoring of plasma stability over time. In addition, an illustration of the optimization of the echelle's aperture plate and photomultiplier tube positions in the focal plane is presented.*

### Introduction

A variety of information is useful in attempting to investigate and improve the analytical capabilities of a new spectroscopic plasma emission source. For instance, common analytical figures of merit include determination of detection limits, linear dynamic range, short- and long-term precision, and susceptibility to chemical interference effects. In addition to these common analytical figures of merit, several researchers [1–5] have emphasized the need for spatial information about a plasma under varying experimental conditions, such as gas flow rates and input power. Spatial information is also useful for evaluating the effects of matrix components in a sample on the analytical capabilities of a plasma [6–9].

While spatial and precision studies are useful in the evaluation of a new plasma emission source, it is often difficult or cumbersome to obtain this type of information with a commercially available spectrometer. Commercial systems are often designed more for the purpose of analysing samples on a routine to semi-routine basis rather than for more specialized, diagnostic studies. As with other researchers [10], the authors have also found that the data analysis and hard-copy output options of commercial spectrometers are often very limited.

After the completion of an experiment, it is very common for the analyst to plot the results. The authors' early work utilizing post-experiment plotting often revealed a flaw or an unexpected result in the experiment that perhaps could have been corrected during the experiment if real-time plotting could have been done. Therefore, another desirable capability to aid in the characterization of a new plasma source is a means of real-time display of acquired data in the form of plots to a graphics screen.

This paper describes the interfacing of an IBM-PC compatible microcomputer to a high-resolution echelle spectrometer to facilitate a variety of data acquisition functions. An integrated, menu-driven program is presented for the purposes of instrument control, data acquisition, real-time plotting, post-experiment analysis, and hard-copy output. This program was developed using ASYST, a commercially available data acquisition and analysis programming environment. The application of this system to the characterization of a new plasma emission source will be described.

### Instrumentation

A schematic representation of the instrumentation is shown in figure 1. The spectrometer, the computer and its peripherals, and the ASYST software will each be described separately. The plasma emission source has been described previously [11–13], and the instrumentation aspects of the source will not be covered here.

### *Spectrometer*

A PLASMA-SPEC (Revision C Board, Leeman Labs, Lowell, Massachusetts, USA) high-resolution echelle spectrometer was used to isolate the analytical spectral line of interest. The merits of echelle spectrometers for high temperature, line-rich emission sources, such as plasmas, have been described [14]. The most frequently cited of these merits is the high resolution attainable. A particularly unique feature of the PLASMA-SPEC spectrometer is the fixed nature of the two dispersion devices (a grating and a prism). Conventional spectrometers generally move a single dispersion device (a grating) to select which wavelength will fall on a fixed detector. Indeed, previous echelle spectrometers involved the

* Present address: Hewlett-Packard Company, Ink-Jet Components Division, 1020 NE Circle Blvd, Corvallis, Oregon 97330, USA.
† Author to whom correspondence should be sent.

0142–0453/91 $3.00 © 1991 Taylor & Francis Ltd.

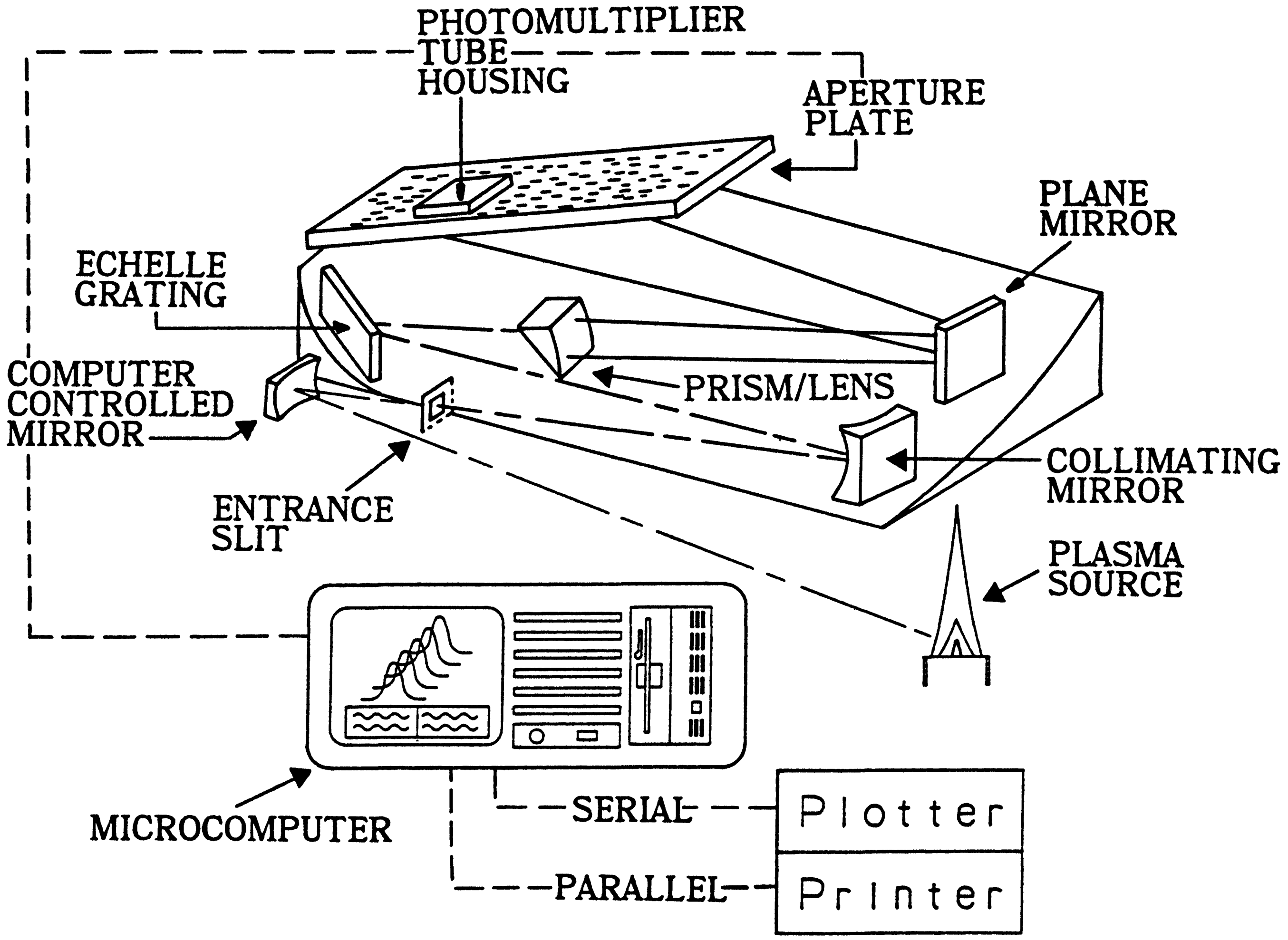

*Figure 1. Schematic representation of instrumentation. Image of plasma source is focused onto entrance slit of echelle spectrometer by a computer-controlled source mirror. Data from the spectrometer are sent to an IBM-PC compatible microcomputer via an RS-232 line. Hardcopy plots are output to a plotter and numeric data to a printer. (Figure adapted with permission from ICP/ECHELLE brochure, Leeman Labs, Inc., Lowell, Massachusetts.)*

movement of their dispersion devices to select lines of interest [15, 16].

The PLASMA-SPEC system utilizes fixed dispersion devices. To achieve line selection, an aperture plate located in the focal plane is moved in conjunction with a photomultiplier tube. This aperture plate, shown in figure 1, contains hundreds of exit slits. To select an analytical line, the aperture plate must be positioned so that one of the exit slits is positioned over the point in the focal plane where the wavelength and order of light of interest appears. In addition, the photomultiplier tube (PMT) must be positioned directly above this exit slit.

The PLASMA-SPEC spectrometer contains a Motorolla 68000-based microprocessor which positions the PMT and aperture plate in the focal plane. However, for the present applications, complete control of the movement of the aperture plate and PMT was required. The reasons for this requirement are twofold. First, the ROM of the spectrometer contains the PMT and plate co-ordinates for 230 lines, which includes 66 elements. It was necessary to access other lines which were not known to the spectrometer, that is, whose plate and PMT co-ordinates were not in the ROM of the spectrometer. Secondly, although the PLASMA-SPEC provided a means of peaking the position of the aperture plate periodically, no such means was readily available for the PMT co-ordinates. When the PMT co-ordinates in the ROM of the spectrometer are used, an optimal PMT position is sometimes not achieved with this early version of the spectrometer. Therefore, a method of optimizing the PMT and plate positions in the focal place was needed.

The PLASMA-SPEC contains one RS-232 communications port through which ASCII coded commands can be sent to the spectrometer. In addition, data from the spectrometer can be sent to an external device via this RS-232 port. Many of the commands for the spectrometer are carried out using the spectrometer's own code called P-Code, which will be described in the section on software. Inherent to the design of the PLASMA-SPEC hardware is the collection of 32 individual data points per acquisition request. In other words, each time a request is made for the spectrometer to begin an acquisition, the acquisition set always consists of 32 samplings which are evenly spaced in time. The duration of each data point is determined by the integration time selected, which can vary from 0·31 s/point to 3 s/point.

As shown in figure 1, the PLASMA-SPEC contains a computer-controllable mirror which focuses an image of the emission source on the entrance slit of the spectrometer. By moving this mirror horizontally and vertically, spatial mapping of the emission source can be achieved. The step resolution of this source mirror is 0·21 mm for horizontal motion and 0·34 mm for vertical motion.

### *Computer and peripheral devices*

The PLASMA-SPEC was interfaced to a Corona PPC-2 (Cordata, Thousand Oaks, California, USA), which is an IBM-PC compatible microcomputer. Communication between the spectrometer and the computer was achieved via an RS-232 port on a JRAM-3 multifunction board (Tall Tree Systems, Palo Alto, California, USA) installed in the Corona. In addition to the RS-232 port, the multifunction board also provided an additional parallel port, clock/calendar functions, and additional memory.

A Star Model SD-10 printer (Star Micronics, Irvine, California, USA) was used to print out data both during and after experiments. An HP7475A plotter (Hewlett-Packard, San Diego, California, USA) was used as the output device for plotting purposes.

### *P-Code routines*

Before describing the ASYST software, some understanding of the routines written in the native language of the spectrometer is necessary. P-Code is the programming code used to control the operation of the PLASMA-SPEC echelle spectrometer. The P-Code instruction set consists of 44 operation codes or 'op-codes'. These op-codes are used to control low-level functions such as loading counters and masking bits, as well as higher-level functions such as initiating data collection and serial port data transmission. A complete list of these op-codes, as well as other details of the P-Code system, can be found in the echelle spectrometer operation manual [17].

The execution of these op-codes is totally independent of any external computer system. The op-codes are processed internally by the spectrometer's own microprocessor. The op-codes can be entered into sequential locations in the spectrometer's memory and executed starting at any location using the menu displayed on the screen of the spectrometer. Since any menu selection offered by the spectrometer can be remotely chosen by ASCII commands sent via the RS-232 line, P-Code routines can be executed as though they were special sub-routines of some external program. Indeed, the P-Code routines which were written were generally executed as sub-routines from the ASYST program running on the PC.

A total of nine routines were written. The first four routines are used to perform aperture plate and PMT high-resolution spectral scans in the X and Y directions of the focal plane. These routines are mainly used in conjunction with externally running software to optimize the aperture plate and PMT positions in the focal plane. This external software will be described in the next section. When a given scan is completed, the 32-point data set from the spectrometer is sent via the spectrometer serial port to the external computer system.

A fifth routine collects data at a specific wavelength and order. In other words, no spectral scanning is done. The aperture plate and PMT are stationary during the execution of this routine. As before, when the 32-point data set has been collected, transmission via the spectrometer serial port is initiated. This routine is generally used in conjunction with external software to collect data once an optimal set of aperture plate and PMT coordinates has been determined.

A sixth and seventh routine perform aperture plate X and Y scans, respectively, as do routines 1 and 2. However, these routines cover a wider spectral range than routine 1, and, therefore, are useful in investigating the background around an analytical line and in searching for lines not in the ROM of the spectrometer.

An eighth routine spatially scans the emission source in the horizontal direction by moving the source mirror of the spectrometer. The range of this spatial scan is controlled by selecting the step size of the source mirror motor.

Finally, routine 9 spatially scans the emission source in the vertical direction. The range of the scan is controlled by varying the step size of the motor operation. The primary use of this routine is to determine the location of the top of the outer quartz tube of the plasma source. A sharp minimum in the vertical spatial profile generally occurs at the top of the quartz tube. This allows all subsequent measurements to be referenced relative to the top of the outer quartz tube.

### *ASYST software*

ASYST (Version 1.51, MacMillan Software Co., New York, USA) is a data-acquisition and analysis programming environment based on the FORTH language [18, 19]. The ASYST software package has been reviewed elsewhere [20–23] and a variety of applications in the area of chemsitry have been published [24–26].

All numeric and string manipulations in ASYST are stack oriented. The ASYST environment is a cross between an interpretive environment, where commands can be given and immediately processed, and a compiled environment. Many predefined, system commands, called 'words', exist for numeric, array, string, plotting, I/O, and other manipulations. These words can be executed in the ASYST environment and interpreted immediately, simply by typing the name of the word. Other, user-defined words can be defined based on the system words. These words can then be compiled and saved as an executable file, and this is how a program is constructed.

Two programs were written in ASYST. The major function of the first program, called ACQDATA, is for data acquisition and real-time graphics plotting of data. The second program, called DATAANAL, was used for post-experimental data analysis and hard-copy plotting. Only ACQDATA will be described here. Since ACQDATA is a menu-driven, integrated program, it is natural to approach the discussion of this program by way of the different menu options. The main menu and each main menu selection will be described in a separate section.

*Main Menu*

The main menu of ACQDATA is shown in figure 2. All menu options are assigned to function keys F1–F10. These menu selections appear in a specially defined window on the screen. The location of this menu window is shown in figure 3, which also shows the rest of the screen setup during a typical acquisition run. In addition to reserving a special portion of the screen for the various menus, a special window is devoted to a text screen, where system prompts and other information appear. The entire middle section of the screen, and the upper left portion of the screen, are reserved for real-time plotting of data. The upper right portion of the screen is another text screen which displays a variety of information, such as the mean value, standard deviation, and relative standard deviation of a data set immediately after it is collected.

Finally, the uppermost line of the screen is divided in half and each side serves as a title line of what appears below. The bottom line of the screen is also divided in half. The left side is reserved for displaying error messages and the right side displays the status of various system variables.

With the exception of the first menu selection, all of the main menu choices shown in figure 2 offer submenus. The various submenus are shown in figure 4. Note that main menu selections F2, F8, and F9 are not currently assigned to any operation. This is indicated by 'NOP' for No OPeration. The one main menu selection which does not offer a submenu is the Zero Motor Coords option, which is selected via the F1 key. This selection allows one to zero the horizontal, vertical, or both of the spatial co-ordinates. These spatial co-ordinates refer to the position of the emission source relative to the entrance slit of the echelle spectrometer. When the source mirror of the PLASMA-SPEC is moved under program control, the horizontal and vertical spatial co-ordinates change. ACQDATA keeps track of these changes. The system status line, shown in figure 3, displays these co-ordinates and updates them as required.

*Manual control*

Selecting F3 from the main menu presents the Manual Control Menus shown in figure 4. This mode allows the most freedom and flexibility in acquiring data. When in the manual mode, the cursor keys on the PC keyboard can be used to move the source mirror of the PLASMA-SPEC. Moving the mirror in this way has the effect of changing the portion of the emission source which falls on the entrance slit of the spectrometer. When the source mirror is moved in this way, the horizontal and vertical spatial co-ordinates are kept track of by ACQDATA and updated on the system status line.

The F1 and F2 selections from the Manual Control Menu are the selections which actually initiate data acquisition. They are both completely general modes of acquisition, that is, a data set will be collected irrespective of what the spatial co-ordinates are. A typical use of the manual mode is to move the image of the emission source to the desired position using the cursor keys. A data set is then acquired at that position by depressing F1 or F2. Movement of the emission source image and subsequent data collection can proceed in this manner indefinitely. When each 32-point acquisition in this manner is finished, the user is prompted for information regarding the optional writing of data to a disk file.

The two manual acquisition keys, F1 and F2, differ in the following manner. When F1 is used, both the aperture plate and PMT are stationary during the acquisition. Therefore, all of the data are at a single wavelength (and grating order). When F2 is used, the aperture plate actually moves in the wavelength direction (dimension) of the focal plane. In this way, a spectral scan is achieved, limited by the aperture slit in the PMT housing.

Selecting F3 provides the ability to move the source mirror by a large amount in the horizontal direction. The horizontal spatial co-ordinate is adjusted accordingly. F4 allows editing of the current background correction mode. This background correction parameter will be discussed later. F5 causes the execution of a P-Code routine which scans the plasma spatially in the horizontal direction and moves to the peak emission point when done. F8 allows changing of the step size of the source mirror, that is, the distance which the source mirror will move each time a cursor key is depressed. The horizontal and vertical step sizes can be different. The F9 selection allows one or both of the spatial co-ordinates to be zeroed. Note that this does not have any effect on the position of the source mirror; only the numbers assigned to the numerical co-ordinates are set to zero. This is useful, for instance, when one has found the peak horizontal position at a particular vertical position in the plasma. Zeroing the horizontal co-ordinate then provides a convenient reference point in the plasma. Finally, F10 returns to the main menu.

*Data Acquisition Menu*

Selecting F4 from the Main Menu brings up the Data Acquisition Menu, shown in figure 4. Apart from the F10 selection, which returns the user to the Main Menu, there are two options. F1 puts the system in the timed acquisition mode. In this mode, data sets, consisting of 32 individual samplings/data set, are collected at periodic intervals. The lengths of these intervals depends on the integration time which is being used.

```
-------------------------Main Menu-------------------------
F1 - Zero Motor Coords       F6 - Echelle Util
F2 - NOP                     F7 - Echelle Set-Up
F3 - Manual Control          F8 - NOP
F4 - Data Acquisition        F9 - NOP
F5 - Optimize Plate/PMT      F10- This Menu
```

*Figure 2. Main menu of ACQDATA.*

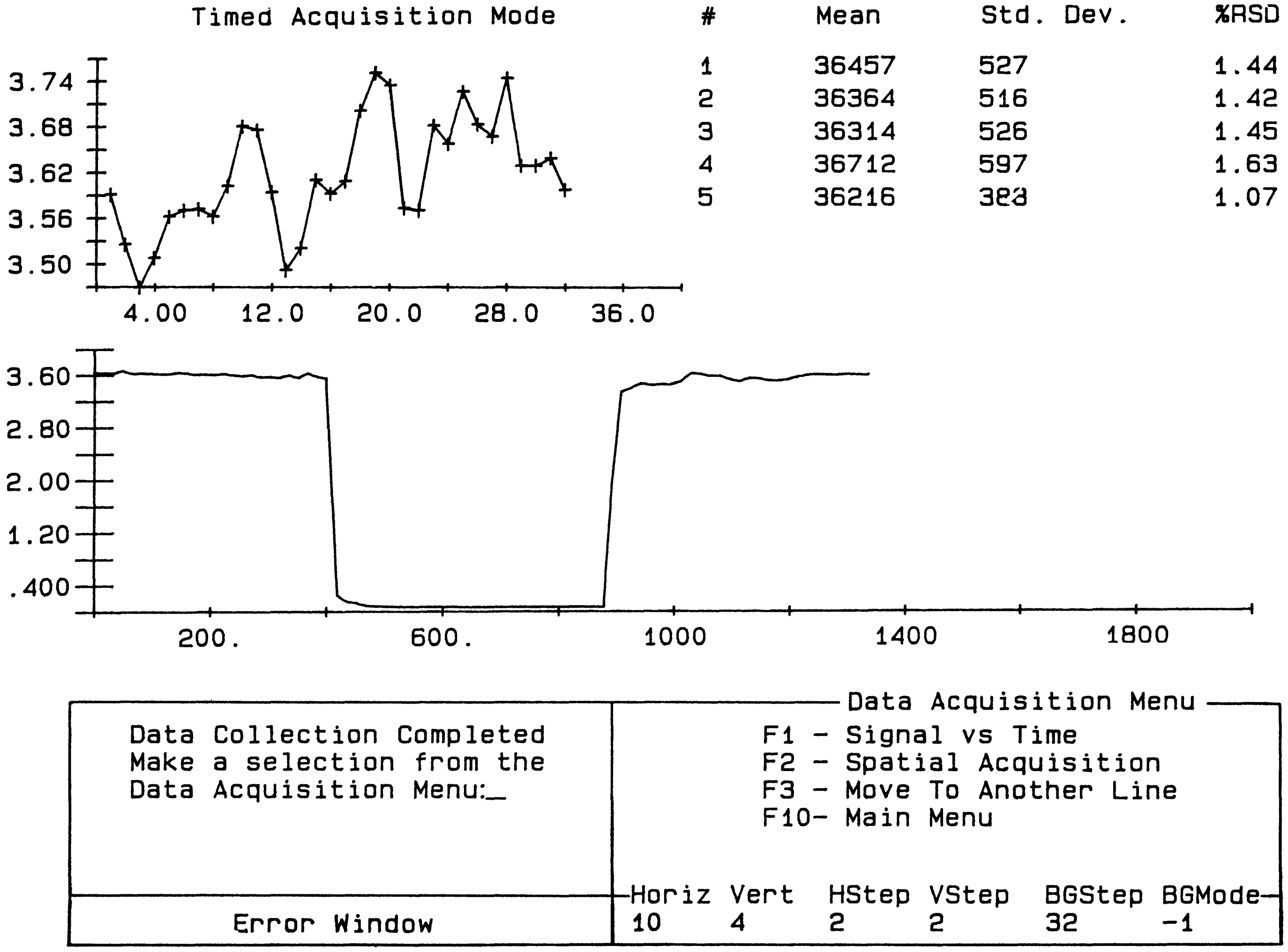

*Figure 3. Example of screen during a timed acquisition experiment. Top left graphics screen shows most recent 32-point data set. Middle graphics screen shows cumulative 32-point average values over time. Lower portion of screen shows menu window, prompt/message window, status line, and error window. Top right of screen displays information about the five most recent acquisition sets.*

Before actually beginning an acquisition session in the timed mode, the user is prompted for information, such as whether to enable real-time plotting. Each raw 32-point data set can be optionally plotted immediately after it is collected. In addition, the average value of each 32-point data set can be plotted versus the time of acquisition of the data set. By default, each 32-point data set is averaged and the standard deviation and relative standard deviation is computed and displayed in the upper right portion of the screen. Figure 3 gives an example of the screen during a timed acquisition session. As can be seen, the raw 32-point values are plotted in the upper left portion of the screen, the average value of each 32-point data set is plotted in the middle, and the statistical values are plotted in the upper right.

During acquisition in the timed mode, the user can optionally halt acquisition and then either continue or exit the acquisition mode. Upon exiting, prompts appear in the text window which allow the data to be written to a disk file. This timed mode of acquisition is particularly useful for investigating such phenomena as the short- and long-term precision of the emission source. This mode is also useful for observing the response of the plasma source to various changes to the system such as sample change-over or a change in a gas flow rate. In general, it is not of interest to move the source mirrors during the timed mode of acquisition. Each 32-point data set is collected by viewing the same portion of the plasma.

The other acquisition mode accessible from the Data Acquisition Menu is geared towards the collection of spatial information and is entered by selecting F2. The function of this mode of acquisition is to successively collect horizontal profiles of the plasma at progressively higher points in the plasma. When the Spatial Acquisition Mode is selected, a variety of user prompts are displayed in the text window. These prompts ask for information such as the desired spatial width of each horizontal profile, the distance to step up vertically after each horizontal profile has been completed, and the total number of horizontal profiles to collect.

As with the Signal versus Time Mode of acquisition, plotting of each 32-point raw data set can be enabled or disabled. However, the spatial mode of acquisition differs from the timed mode in that during the collection of each 32-point data set, the source mirror is stepping in the horizontal direction. Therefore, each one of the 32 data points corresponds to a different horizontal position in the

```
Manual Control Menu
Use Cursor Keys To Move Mirror OR
F1 - Acq Data Point        F6 - NOP
F2 - Acq Wave Scan         F7 - NOP
F3 - Gross X Move          F8 - Edit Step Size
F4 - Edit Bkgd. Mode       F9 - Zero Coords
F5 - Peak Horiz. Pos.      F10- Main Menu
```

```

Data Acquisition Menu

F1 - Signal vs Time

F2 - Spatial Acquisition

F10- Main Menu

```

```

Optimization Menu

F1 - Plate X

F2 - Plate Y

F3 - PMT X

F4 - PMT Y

F10- Main Menu

```

```
Echelle Utilities Menu
F1 - Ed/Create Coord Set F6 - NOP
F2 - Change # Coord Sets F7 - Print Coord Set
F3 - Coord Set To File   F8 - NOP
F4 - Coord Set From File F9 - NOP
F5 - Lst Coord Set Files F10- Main Menu
```

```
Echelle Set-Up Menu
F1 - Down Load P-Code
F2 - Load Hg Mirror Motor Op
F3 - Set Integration Time
F4 - Edit Background Offset
F5 - Load Hg PMT Coordinates
F6 - Quartz Finder F10-Main Menu
```

*Figure 4. Submenus of ACQDATA.*

plasma. So if the plotting of each 32-point data set is enabled, one can view the horizontal profile at a particular vertical position in the plasma immediately after the data are collected.

Another pre-acquisition prompt requests the type of background correction to be used. One or two-point off-line background corrections can be selected or the background correction can be done by introducing a blank solution to the plasma and repeating the spatial acquisition under the same conditions. If the off-line type of background correction is selected, the background information is collected immediately after each horizontal profile. However, the analyte emission data and the background data consist of independent 32-point data sets.

Finally, the user can optionally interrupt acquisition after each horizontal profile has been completed. At this point, acquisition can be restarted or the user can exit. When the user exits in this manner, or when all of the requested profiles have been completed, prompts appear which request the necessary information for saving the data to a disk file.

*Optimize plate/PMT*

The fifth choice from the Main Menu facilitates the optimization of the position of the aperture plate and PMT in the focal plane of the echelle. Selecting F5 presents the Optimization Menu, shown in figure 4. Selecting F1 through F4 from the Optimization Menu initiates a peaking routine which scans the aperture plate (or PMT) over a very small distance in the horizontal or vertical dimension of the focal plane. Then, an algorithm in ACQDATA determines a correction factor. This correction factor represents the amount by which the aperture plate (or PMT) must be moved from its current position to achieve maximum light throughput. The user can enable or disable the display of the aperture plate and PMT scans. In addition, the user has the option, after seeing what the scan looks like, of applying the correction factor. If the correction factor is applied, a library of aperture plate and PMT co-ordinates is updated by ACQDATA for the particular line in use. This library system is described below.

*Echelle Utilities Menu*

Selecting F6 from the main menu presents the Echelle Utilities Menu shown in figure 4. These menu selections allow the user to manipulate a library of aperture plate and PMT co-ordinates. To set the spectrometer to a wavelength in a particular order of the grating, a unique set of four co-ordinates is required: the horizontal and vertical aperture plate co-ordinates and the horizontal and vertical PMT co-ordinates. From the Echelle Utilities Menu, the user can create or edit a co-ordinate set (F1) and save it to a file (F3). At a later time, the desired co-ordinate set can be retrieved (F4) and made known to the system. In this way, the co-ordinate sets need not be manualy re-entered each time an experiment is performed. The desired co-ordinate set can simply be retrieved from the library file. F5 lists, to the monitor, the elements whose co-ordinates are in the library. F7 prints the actual co-ordinates for a particular wavelength and order to either the monitor or the printer. Finally, F2 allows the user to change the number of co-ordinate sets which the system will keep track of.

*Echelle Set-Up Menu*

The final choice offered by the Main Menu (F7) presents the Echelle Set-Up Menu shown in figure 4. These selections allow the user to perform a variety of tasks which are required before an experiment can begin. For instance, the first choice, F1, automatically down-loads the P-Code routines to the spectrometer. These P-Code routines, which were discussed previously, need to be reloaded occasionally due to the occurrence of a power outage, a complete power-down of the spectrometer for maintenance purposes, or if another user down-loads different P-Code routines to the spectrometer. The F2 and F5 selections relate to an internal Hg lamp which can be used for wavelength calibration purposes. F3 allows the integration time to be set to a value other than the default value. F4 allows the value of the background offset to be edited. This background offset determines how far off-line the background correction will be made. Finally, F6 initiates a routine which 'finds' the top of the quartz tube by looking for the sharp discontinuity in the vertical emission profile caused by the quartz tube. The top of the quartz tube can then be used as a reference point for all subsequent vertical positions. As is the case throughout all of the menus, F10 will again present the Main Menu.

### Application

*Optimization of aperture plate and PMT positions in focal plane*

As was mentioned previously, there is a need to optimize the position of the aperture plate and the PMT in the focal plane. Both the aperture plate and the PMT can vary in two directions. An example of how a PMT optimization proceeds is as follows. When F3 is selected from the Optimization Menu, the PMT is moved over a very narrow distance in the wavelength domain and intensity readings are taken throughout the scan. A typical profile before optimization is shown in figure 5(*a*). The optimization algorithm uses the width at half peak maximum to determine the centre of the profile. After this initial profile is obtained, ACQDATA produces a correction factor to properly centre the profile, applies it after user confirmation, reruns the scan, and displays a corrected profile, which is shown in figure 5(*b*). A similar procedure occurs for the other optimization selections in the Optimization Menu.

The consequences of a non-optimal PMT position are twofold. First, when the PMT co-ordinates for a given wavelength and order in the ROM of the spectrometer are used, a profile of the type shown in figure 5(a) may result. During a typical experiment, the PMT would be fixed at the position marked P throughout the data acquisition period. Therefore, since the Y axis of the profile in figure 5(*a*) represents emission intensity, it is evident that the maximum possible amount of light is not being collected by the PMT. This can degrade detection limits and sensitivity.

Secondly, if the PMT is at the position marked P in figure 5(*a*), as it is when the ROM values of the spectrometer are used to position the PMT, increased noise will result due to slight changes in the PMT position. This is due to the positioning of the PMT on the side of the profile instead of on the plateau region of the profile. However, after optimization of the PMT position (figure 5[*b*]), the PMT is positioned at the plateau of the profile. Therefore, a slight change in the PMT position will not significantly change the signal intensity. Slight changes in the PMT position can occur over time due to such factors as fluctuation in the room temperature.

*Monitoring of plasma stability*

The Signal versus Time Mode, which is selected from the Data Acquisition Menu (figure 4), can be useful for monitoring the stability of the plasma over time. Emission resulting from the introduction of 100 μg/ml of Zn into the plasma was monitored for a period of 360 s. Figure 6 shows the results of this Signal versus Time acquisition session. The small dots (·) represent the individual 32 points in each data set while the ◆ symbols represent the average value of each 32-point data set taken over the time period.

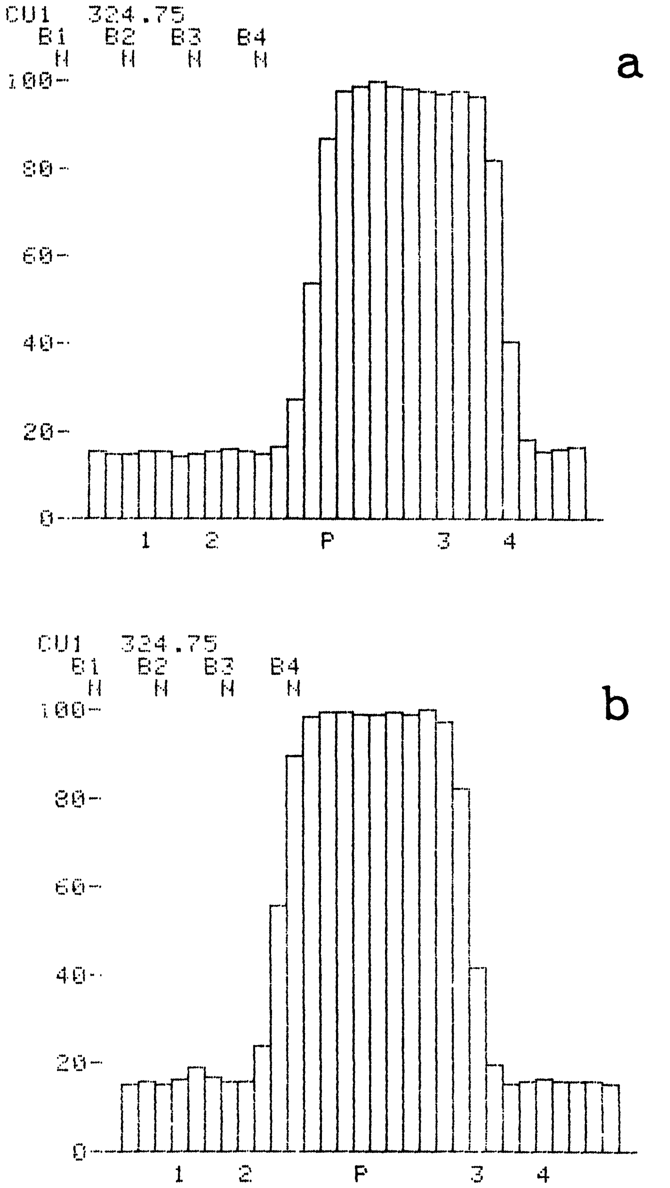

*Figure 5. (a) Aperture plate scan at the CuI 324·75-nm line before optimization. The profile should be centred about the 'P' position for maximum light throughput. (b) Aperture plate scan at the CuI 324·75-nm line after the ACQDATA plate optimization routine has been performed. Profile is now centred about the 'P' position where maximum light throughput and minimum noise occur.*

This graph is displayed on the computer screen and updated with each additional 32-point data set as it is collected. In addition, as shown in figure 3, the mean, standard deviation, and relative standard deviation of each 32-point data set are displayed.

This mode of acquisition provides a useful means of monitoring drift in the system. Since the time interval between data sets can easily be changed, short or long-term drift can be monitored. In addition, this timed mode of acquisition provides an excellent means of watching the effect of a change in an experimental parameter on the

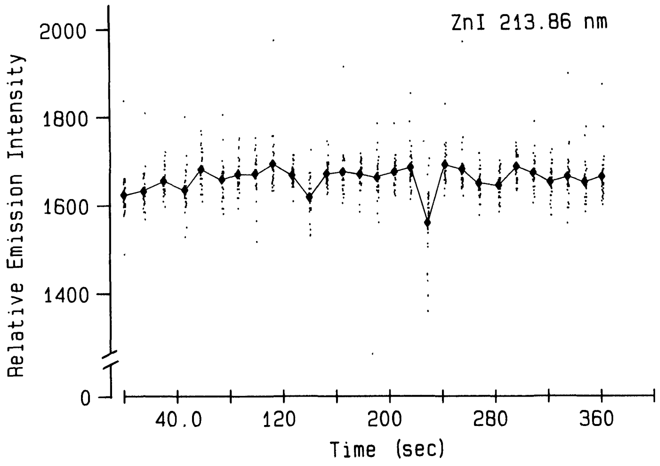

*Figure 6. ZnI emission at 213·86 nm versus time. Monitoring of plasma stability using the timed acquisition mode of ACQDATA. The small dots (·) represent the individual points of each 32-point data set. The solid line and symbol (◆) represent the average value of each 32-point data set.*

signal. In the present application, this mode of acquisition was frequently used to observe the effect of a change in a plasma gas flow on the plasma emission intensity.

*Monitoring of nebulizer/spray chamber wash-out time*

Figure 7 illustrates how the emission intensity changes with time after a blank solution is introduced into the system. The original sample concentration was 100 μg/ml of Ca. As in the previous example, the Signal versus Time Mode was used. This provides an excellent way of measuring the time required for sample change-over in the nebulizer system.

*Spatial profiles of the plasma*

The Spatial Acquisition Mode, which is selected from the Data Acquisition Menu, facilitates the acquisition of spatial information about the emission from the plasma

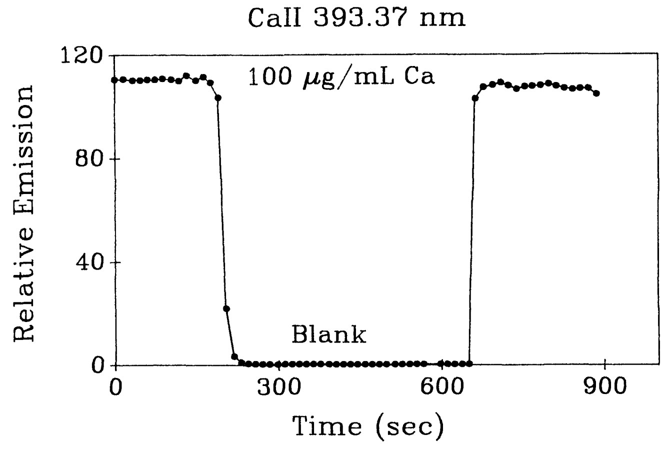

*Figure 7. Monitoring of nebulizer wash-out period upon change-over from 100 μg/ml Ca to blank. Timed acquisition of ACQDATA was used. Monitoring CaII emission at 393·37 nm. Each point on the plot represents the average of 32-data points taken over a time period of 1·5 s.*

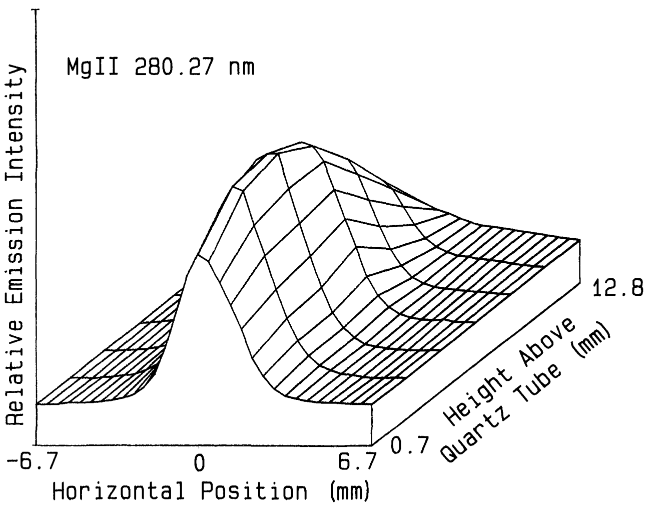

*Figure 8. Horizontal emission profiles of plasma at seven different observation heights above outer quartz tube. Monitoring the MgII 280·27 nm line while aspirating a 10 μg/m solution.*

source. Figure 8 shows a set of horizontal emission intensity profiles, each taken successively at a different vertical height in the plasma. All vertical positions are referenced from the top of the outer quartz tube of the plasma assembly. This particular set of horizontal profiles was obtained while a solution of 10 μg/ml of Mg was introduced to the plasma. The ion line of Mg at 280·27 nm is being monitored.

Spatial information of the type provided by the spatial acquisition mode is useful in the investigation of a variety of experimental variables. Figure 8 shows that the emission profile is highly symmetrical in the horizontal direction and that there is a height above the quartz tube that is optimal for viewing the emission intensity. This spatial emission profile, however, is highly sensitive to a variety of experimental parameters such as nebulizer gas flow, height of the sample introduction tube, plasma electrode heights, and current to the plasma [11]. Thus, this mode of acquisition provides an easy way of visually observing, in real-time, the effects of these experimental variables on the spatial emission profiles.

### Conclusions

The software presented here provides a means of acquiring data of the type that is often very difficult to obtain with the capabilities of many commercially available spectrometer systems. In addition, the software provides the analyst with real-time feedback of data as it is obtained. This feedback is in the form of plots to a graphics screen of the computer monitor and is useful in monitoring the course of an experiment in progress. This real-time feedback offers an advantage over the common post-experiment feedback in that it allows the analyst to alter the experiment in progress or modify an experimental variable to improve subsequent results.

The aperture plate and PMT optimization features of the software allow the analyst to fine tune the position of the plate and PMT in the focal plane of the spectrometer. This results in an increase in the amount of light to the detector thus potentially improving detection limits and sensitivity.

Perhaps most importantly, fundamental studies of a plasma source, which often require a knowledge of the spatial emission characteristics of the plasma, are facilitated by the flexible spatial acquisition mode. This mode automates the acquisition and display of real-time horizontal and vertical emission profiles of the plasma source.

### Acknowledgements

This work was supported in part by the National Science Foundation, Grant Number CHE-8206692. The authors also gratefully acknowledge the support of Leeman Labs, Inc., Lowell, Massachusetts, USA. The reception of an N. L. Tartar Fellowship by one of the authors (J. P. S.) is also gratefully acknowledged.

### References

1. Boumans, P. W. J. M. and de Boer, F. J., *Spectrochimica Acta* (1976), **31B**, 355.
2. Kornblum, G. R. and de Galan, L., *Spectrochimica Acta*, **32B** (1977), 455.
3. Edmonds, T. E. and Horlick, G., *Applied Spectroscopy*, **31** (1977), 536.
4. Horlick, G. and Blades, M. W., *Applied Spectroscopy*, **34**, (1980), 229.
5. Blades, M. W. and Horlick, G., *Spectrochimica Acta*, **36B** (1981), 861.
6. Savage, R. N. and Hieftje, G. M., *Analytical Chemistry*, **52** (1980), 1267.
7. Blades, M. W. and Horlick, G., *Spectrochimica Acta*, **36B** (1981), 881.
8. Rybarczyk, J. P., Jester, C. P., Yates, D. A. and Koirtyohann, S. R., *Analytical Chemistry*, **54** (1982), 2162.
9. Faires, L. M., Apel, C. T. and Niemczyk, T. M., *Applied Spectroscopy*, **37** (1983), 558.
10. Hites, B. R., Olesik, J. W., Parisi, A. F. and Hieftje, G. M., *Computer Enhanced Spectroscopy*, **3** (1986), 91.
11. Shields, J. P., Lee, G. H. and Piepmeier, E. H., *Applied Spectroscopy*, **42** (1988), 684.
12. Shields, J. P. and Piepmeier, E. H., *Applied Spectroscopy*, **42** (1988), 693.
13. Shields, J. P., Ph.D. thesis. Department of Chemistry, Oregon State University, Corvallis, Oregon (1987).
14. Keliher, P. N. and Wohlers, C. C., *Analytical Chemistry*, **48** (1976), 333A.
15. Elliot, W. G., *American Laboratory*, **2**(3) (1970), 67.
16. Matz, G. J., ibid., **5**(3) (1973), 75.
17. *PLASMA-SPEC Operations Manual*, Leeman Labs, Inc., Lowell, Massachusetts, USA.
18. Moore, C. H., *Byte*, **5**(8) (1980), 76.
19. Reynolds, T. J., *Journal of Microcomputer Applications*, **8** (1985), 255.
20. Bellman, Jr., R. A., *Research and Development*, February (1988), 100.
21. Borman, S. A., *Analytical Chemistry*, **57** (1985), 983A.
22. Hary, D., Oshio, K. and Flanagan, S. D., *Science*, **236** (1987), 1128.
23. Eckhouse, R., *Computer*, March (1989), 81.
24. Keller, J. W., Gould, T. F. and Aubert, K. T., *Journal of Chemical Education*, **63**(8) (1986), 709.
25. Chapman, D. W. and Shabushnig, J. G., *Instruments and Computers*, March/April (1986), 83.
26. Mannino, S., Fregapane, G. and Bianco, M., *Electroanalysis*, **1** (1989), 177.

